# Endoscopic resection combined with the Cryoballoon focal ablation system in the porcine normal esophagus: a preclinical study

**DOI:** 10.1186/s12876-021-01819-0

**Published:** 2021-05-22

**Authors:** Hironori Sunakawa, Yusuke Yoda, Nobuyoshi Takeshita, Hiro Hasegawa, Kenji Takashima, Tomohiro Kadota, Takeo Fujita, Tetsuo Akimoto, Satoshi Fujii, Tomonori Yano

**Affiliations:** 1 Department of Gastroenterology and Endoscopy, National Cancer Center Hospital East, 6-5-1, Kashiwanoha, Kashiwa, 277-8577 Japan; 2 NEXT Medical Device Innovation Center, National Cancer Center Hospital East, Kashiwa, Japan; 3 Department of Colorectal Surgery, National Cancer Center Hospital East, Kashiwa, Japan; 4 Division of Esophageal Surgery, National Cancer Center Hospital East, Kashiwa, Japan; 5 Course of Advanced Clinical Research of Cancer, Juntendo University Graduate School of Medicine, Bunkyo-ku, Tokyo, Japan; 6 Department of Radiation Oncology and Particle Therapy, National Cancer Center Hospital East, Kashiwa, Chiba Japan; 7 Division of Pathology, Exploratory Oncology Research and Clinical Trial Center, National Cancer Center, Tokyo, Japan

**Keywords:** Combination treatment, Cryoablation, Endoscopic resection, Esophageal neoplastic tissue

## Abstract

**Background:**

The Cryoballoon focal ablation system (CbFAS) for dysplastic Barrett’s esophagus is simple, time-saving and has high therapeutic efficacy. This study aimed to evaluate the technical feasibility and tissue damage with combination therapy of endoscopic resection (ER) and CbFAS in porcine models.

**Methods:**

Three pigs (A, B, and C) were included, and all ER procedures were performed by endoscopic mucosal resection using the Cap method (EMR). Combination therapy for each pig was performed as follows: (a) CbFAS was performed for a post-EMR mucosal defect for Pig A; (b) CbFAS for post-EMR scar for Pig B, and (c) EMR for post-CbFAS scar for Pig C. All pigs were euthanized at 32 days after the initial procedure, and the tissue damage was evaluated.

**Results:**

All endoscopic procedures were followed as scheduled. None of the subjects experienced anorexia, rapid weight loss, bleeding, and perforation during the observation period. They were euthanized at 32 days after the initial endoscopic procedure. On histological assessment, there was little difference between the tissue that was treated with CbFAS alone and that treated with CbFAS in combination with ER.

**Conclusion:**

Combination therapy with ER and CbFAS can be technically feasible, and its outcome was not significantly different from CbFAS alone in terms of tissue damage.

**Supplementary Information:**

The online version contains supplementary material available at 10.1186/s12876-021-01819-0.

## Introduction

Endoscopic resection (ER), including endoscopic mucosal resection (EMR) and endoscopic submucosal dissection (ESD), is widely accepted as a minimally invasive treatment for superficial esophageal squamous cell carcinoma (SESCC) [1]. Although there are cases of patients undergoing curative resection with ER, there exists a risk for developing multiple, metachronous or recurrent SESCC in the preserved segment of the esophagus [2]. Furthermore, these SESCC lesions may sometimes develop near the ER scar and pose challenges to ER because of postoperative submucosal fibrosis due to the previous ER. Another concern is that patients who have been treated with ER especially for a large SESCC or with repeated ER for multiple SESCC have an increased risk for esophageal strictures [3, 4].

The Cryoballoon focal ablation system (CbFAS; C2 Cryoballoon, HOYA Pentax Medical, Japan) has increasingly received attention as a novel device for the ablation of esophageal neoplastic tissue. Preliminary clinical studies in patients with Barrett’s esophagus have shown that CbFAS is a simple, safe, and effective procedure for the removal of dysplastic Barrett’s esophagus and esophageal squamous cell neoplasia [5–7]. Cryoablation works by making a cold ablations injury to the cells in the tissue while preserving the collagen matrix architecture. It differs from tissue-heating ablations such as radiofrequency ablation (RFA), which sometimes develop the fibrosis or stricture of esophageal lumen. Therefore, cryoablation is expected to have the potential to facilitate deeper ablation with lower stricture rates [8–10]. Thus, CbFAS may be a promising therapeutic option in combination with ER for lesions having a risk of esophageal stenosis due to ER or technical difficulty of ER. However, there is limited data on combination therapy with ER and CbFAS for SESCCs.

This study was conducted to evaluate the feasibility and safety of procedures and pathological tissue damage in combination therapy with ER and CbFAS in a porcine model. In this study, three combination treatments were planned to provide treatment options for residual lesions after EMR, residual lesions after CbFAS, and lesions suspected to have residual lateral or vertical margins during ER.

## Methods

### Experimental animals and study protocol

This study was conducted at the National Cancer Center Hospital East, Kashiwa, Japan. The study protocol was approved by the Animal Experiment Committee in National Cancer Center JapanK18-024, 2018/12/27), and all animal experiments were conducted in accordance with the Institutional guidelines and the ARRIVE guidelines (http://www.nc3rs.org.uk/page.asp?id=1357). We included three female pigs (weight 40–45 kg). The study protocol was designed to minimize pain or discomfort to the animals. To evaluate the feasibility of combination therapy in the various situation, we created four lesions on each of the three porcine normal esophagi using a DualKnife™ (Olympus, Tokyo, Japan). We employed three combination therapy strategies: (a) simultaneous procedure of EMR and CbFAS for Pig A, (b) CbFAS for post-EMR scar for Pig B, and (c) ER for post-CbFAS scar for Pig C. All EMR procedures were performed through EMR using the Cap method (EMR).

### Study procedures

**Fig. 1:**
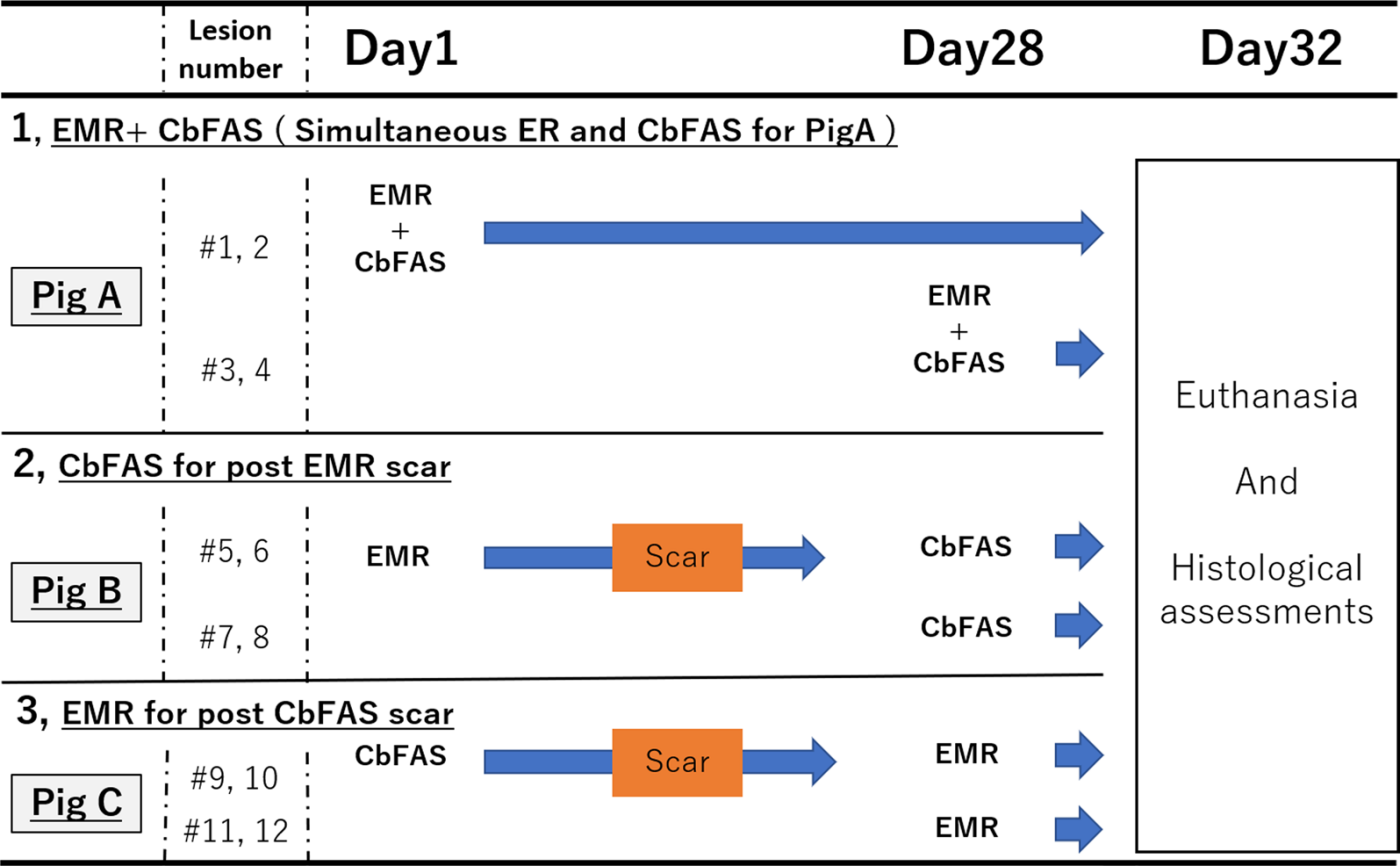
Study flowchart

**Fig. 2:**
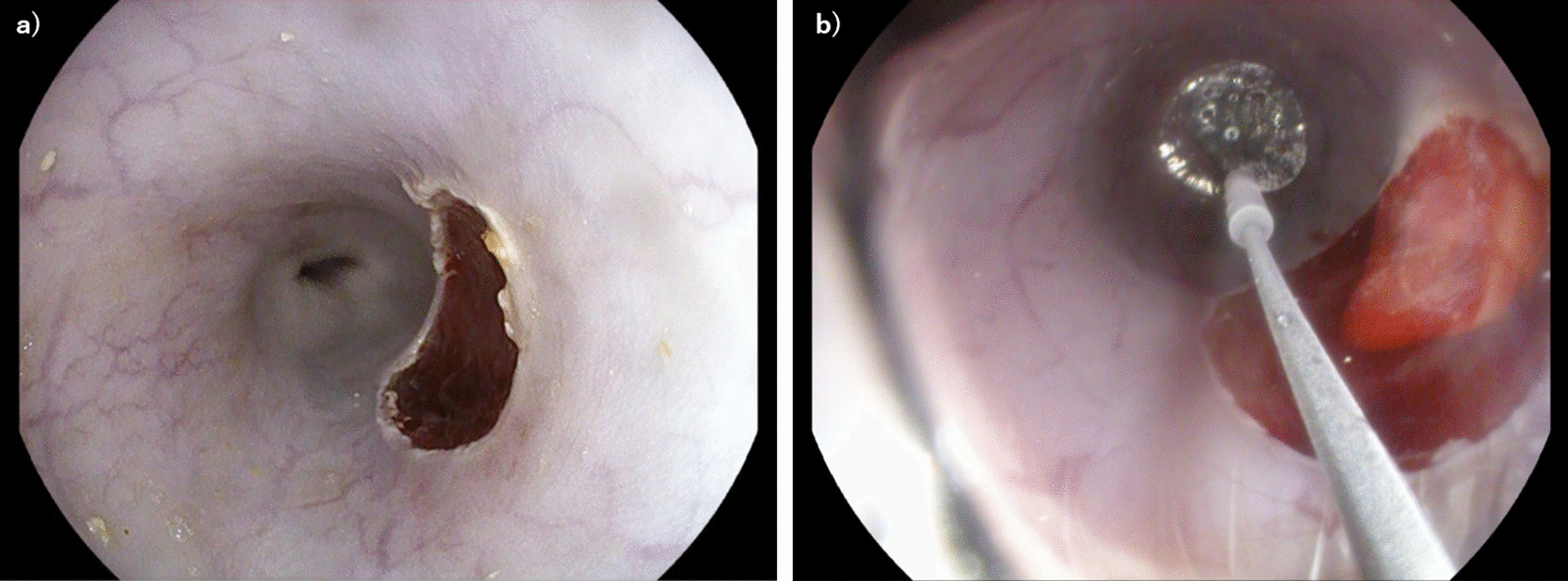
**a** Mucosal defects were seen after endoscopic mucosal resection using the Cap method at the right wall of the esophagus. **b** The Cryoballoon focal ablation system was applied at the mucosal defect site

In vivo porcine specimens were obtained under general anesthesia: sedation was induced with intramuscular midazolam (1 mg/kg) and ketamine (15 mg/kg), and anesthesia was induced and maintained with propofol (3 mg/kg initially, and 6 mg/kg/h, respectively). The details of procedural protocol in each pig were as follows. In Pig A, we simultaneously used EMR and CbFAS (ER + CbFAS; the CbFAS was done immediately after EMR for a post-EMR mucosal defect), wherein EMR + CbFAS were undertaken at two lesions on Day 1 (# 1,2), and at another two lesions on Day 28 (# 3,4) (Fig. 1) (Supplementary Video). In Pig B, we conducted CbFAS for a post-EMR scar; the EMR was done for two lesions on Day 1 (# 5,6), and the CbFAS was carried out on two lesions of EMR scarring as well as at two lesions with normal mucosa on Day 28 after the EMR (# 7, 8) (Fig. 2). In Pig C, we conducted EMR for post-CbFAS scarring; CbFAS was done for two lesions on Day 1 (# 9, 10), and EMR was carried out for two lesions on the post-CbFAS scarring as well as for two normal lesions on Day 28(# 11, 12). All endoscopic procedures were undertaken as scheduled by three surgeons. (HS, YY. TY).

### Cryoablation

We used the CryoBalloon focal ablation system (CbFAS; C2 Cryoballoon, HOYA Pentax Medical, Japan) in this study. The CbFAS comprises a portable hand-held Controller, a catheter with a self-sizing balloon with a spray hole located on a diffuser, foot pedal for adjusting the balloon and the diffuser, and a single-use cartridge of liquid nitrous oxide. The distal tip of the catheter (diameter 3.6 mm) is advanced through a PENTAX EG34-i10 therapeutic endoscope channel (HOYA Pentax Medical, Japan) and the proximal end is connected to the Controller to operate the catheter with the foot pedal. From the cartridge, which is also connected to the Controller, liquid nitrous oxide (− 85 °C) is released through the catheter. By rotating the diffuser clockwise or counterclockwise, the spray hole can be directed to the targeted area. The balloon probe is placed in contact with the tissue wall of the target region, and cryogenic fluid is sprayed while visualizing the target site through the balloon. A single application created an ice patch of approximately 2 cm^2^ on the targeted mucosa. Ablations of 8 s durations were performed in this study.

### Endoscopic mucosal resection using a cap-fitted endoscope

The EMR technique requires a specialized transparent cap that is fitted to the tip of an endoscope. Saline is injected into the submucosa. The crescent-shaped snare (Olympus, Tokyo, Japan) is then pre-looped into the groove of the rim of the cap. Then, the lesion is suctioned with medium to high vacuum into the cap. After the endoscopist strangulates the lesion by closing the snare, electrosurgical current is used to resect the lesion.

### Outcomes

The primary outcome was to evaluate the feasibility of combination treatment. We evaluated the technical success (defined treatment completion as intended) and device malfunction (defined as any failure in CbFAS components requiring device replacement). In addition, the difficulty of each process in all procedures, including injection of saline into the submucosa, lifting the mucosa, and snaring and cutting in ER, and stabilizing the balloon and ablation in CbFAS, was recorded. The degree of difficulty was classified into three levels: easy, moderate, and difficult. Easy was defined by the procedure being performed without any problems, and difficult as instances when the procedure had failed several times and needed to be repeated, or when the treatment was not performed as planned. Moderate was defined as being intermediate in terms of ease and difficulty. In addition, all procedures were evaluated as easy or moderate in the technical success case. The secondary outcome was safety. We evaluated bleeding and perforation during treatment and esophageal stenosis, weight loss/gain, and anorexia during this study period.

### Histopathological analysis

All animals were euthanized by intravenous injection of potassium chloride on days 32 after the initial procedure, and tissue specimens of EMR were harvested for histopathological examination. Histopathological outcomes were evaluated on the basis of tissue damage on days 5 and 32 after treatment. Tissue sections were prepared from samples of the ablated areas and EMR specimens. All specimens were fixed in formalin (10%), embedded in paraffin, and stained with hematoxylin and eosin. Slices of the specimens were evaluated for depth of tissue damage and tissue finding caused by ablation damage in the esophageal wall. All specimens were assessed by a gastrointestinal pathologist.

## Results

### Feasibility

The procedure was technically successful in all combination therapy strategies. Technical difficulty and procedure success rate of each treatment strategy are shown in Table 1. In CbFAS even for EMR scars, the stability of balloon was achieved easily and there was no technical difficulty. In terms of procedure time, there was not difference between EMR VS EMR for CbFAS scar and CbFAS VS CbFAS for EMR scar. In EMR for CbFAS scars, lifting with submucosal injection was not smooth as compared to that in the normal area, but the snaring and cutting was technically easy.

**Table 1:** Technical feasibility of combined ER and CbFAS

	EMR	EMR for CbFAS scar
EMR vs EMR for CbFAS scar		
Injection to submucosa	Easy	Moderate
Mucosal lifting	Easy	Moderate
Snaring and cutting	Easy	Easy
Procedure success rate	2/2	2/2

	CbFAS	CbFAS for EMR scar
CbFAS vs CbFAS for EMR scar		
Stability of balloon	Easy	Easy
Ablation	Easy	Easy
Procedure success rate	2/2	2/2

Easy was defined by the procedure being performed without any problems

Moderate was defined as being intermediate in terms of ease and difficulty

Difficult is instances when the procedure had failed several times and needed to be repeated, or when the treatment was not performed as planned

EMR, endoscopic mucosal resection; CbFAS, Cryoballoon focal ablation system

### Safety

**Fig. 3:**
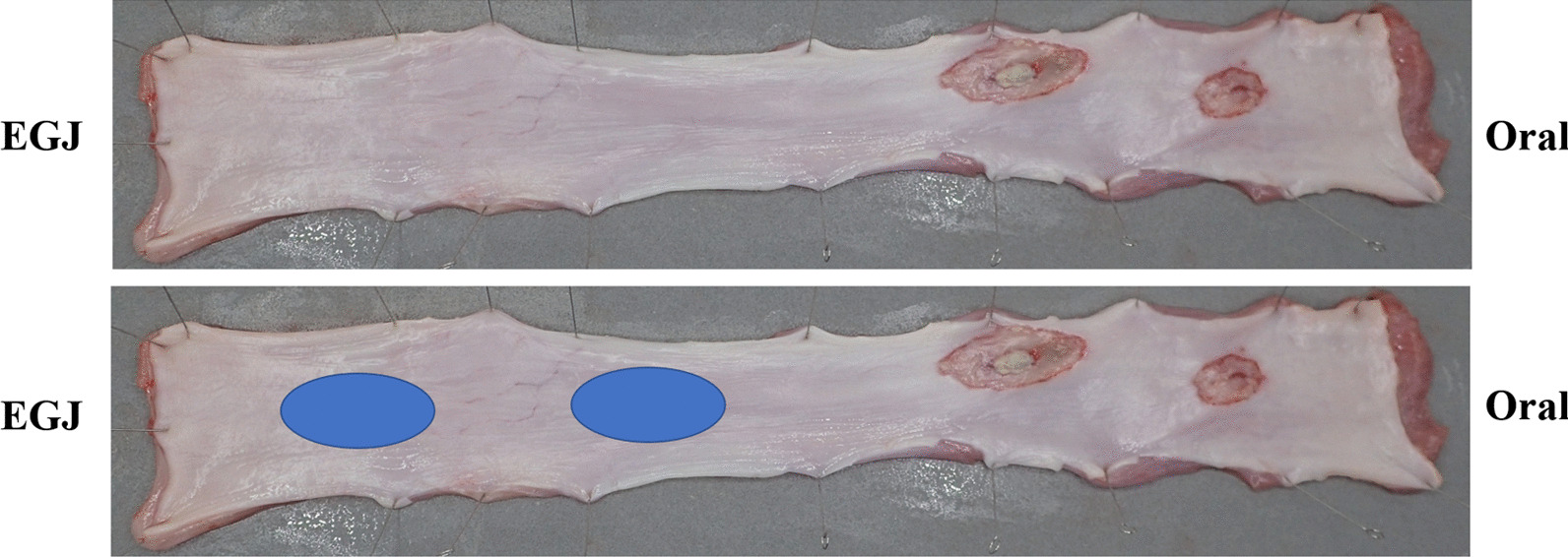
Esophagus after excision. The treatment (Simultaneous use of endoscopic mucosal resection and Cryoballoon focal ablation system) was performed in the blue circle position, but there was no obvious scarring or stenosis

The esophageal diameter was hardly reduced even after ulcer healing (Fig. 3). No device malfunction occurred in this study. None of the study animals experienced anorexia, weight loss (Table 2), bleeding, or perforation during the observation period.

**Table 2:** The weight of pigs during the study

	Day 1 (before) (kg)	Day 7 (kg)	Day 14 (kg)	Day 28 (kg)
Pig A	42.2	42.2	43.5	43.6
Pig B	44.1	44.0	44.8	46.3
Pig C	42.5	42.1	43.4	44.2

### Histological analysis

**Fig. 4:**
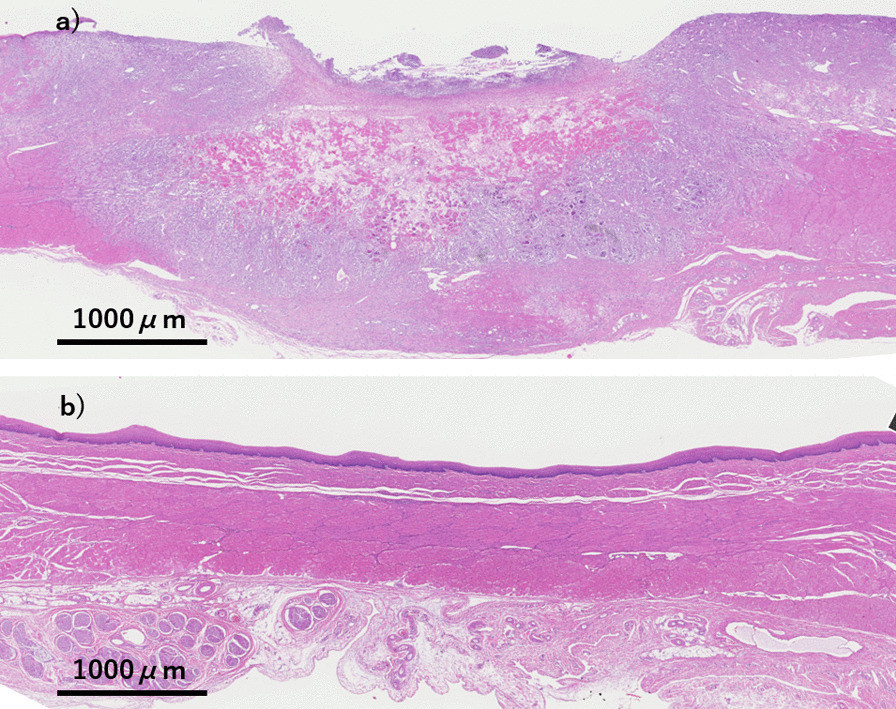
Histological effects at different time frames after esophageal focal cryoablation. **a** The Cryoballoon focal ablation system + endoscopic mucosal resection resulted in necrosis throughout the entire esophageal wall at 4 days after treatment. **b** The treated areas showed only mild fibrosis 32 days after the treatment

Tissue damage of EMR after CbFAS specimens showed only mild fibrosis in the lamina propria mucosae compared to EMR specimens for normal areas. Tissue damage of treatment in pigs A and B are shown in Table 3. There was no difference in tissue damage between CbFAS alone and CbFAS combined with ER. Although the tissue damage of EMR + CbFAS spread all layers of the esophageal wall at 4 days after treatment, only fibrosis was observed in the submucosa at 32 days after treatment (Fig. 4).

**Table 3:** Pathological evaluation of treatment in pigs A and B

	Lesion number	Treatment	Time to euthanasia from treatment (days)	Pathological evaluation		
				Size of EMR ulcer (mm)	Depth of tissue damage	Findings of ablation damage
Pig A	#1	EMR + CbFAS	32		Submucosa	Fibrosis
	#2				Submucosa	Fibrosis
	#3		4	33 × 14	Muscle layer (inner circular layer)	Necrosis
	#4			22 × 10	Adventitia	Necrosis
Pig B	#5	CbFAS for EMR scar	4		Muscle layer (inner circular layer)	Necrosis
	#6				Submucosa	Necrosis
	#7	CbFAS alone			Adventitia	Necrosis
	#8				Submucosa	Necrosis
Pig C	#9	EMR for CbFAS scar	4	18 × 12	Submucosa	
	#10			20 × 10	Submucosa	
	#11	EMR		25 × 10	Submucosa	
	#12			32 × 15	Submucosa	

EMR, endoscopic mucosal resection; CbFAS, Cryoballoon focal ablation system

EMR + CbFAS, simultaneous use of ER and CbFAS

## Discussion

In this study, we first assessed the technical feasibility and tissue damage of combined ER and CbFAS in an animal model. In the ER to the area after CbFAS, the ER procedure was successful even in cases with some unsteady flow and mucosal swelling during the saline injection into submucosa at the site of the minor scar after the CbFAS. Moreover, there were no major histological differences between the tissue of with ER followed by CbFAS or CbFAS alone. These results indicated that the combination of ER and CbFAS is feasible and is a promising treatment strategy for esophageal neoplasms.

We could comparatively evaluate the tissue damage due to EMR + CbFAS (simultaneous ER and CbFAS) between the acute and delayed phase in the same subject. The difference of findings was valuable because there have been only a few studies evaluating this method whereby a CbFAS for mucosal defects is performed immediately after the EMR. These results are similar to the pathological findings after CbFAS alone of previous animal studies [11]. In addition, there were no complications during the follow-up period, even when the deep tissue damage extended into the adventitia in the acute phase in the present study. In the previous porcine animal studies of RFA for the mucosal defect after an ER, two delayed perforations (11%) occurred, and they concluded that single-step treatment with EMR and RFA was not recommended in the clinical practice [12]. A key feature of cryoablation is that it does not disrupt the extracellular matrix, unlike RFA [13], and may reduce complications such as perforation, despite the tissue damage it causes.

We used an ablation duration of 8 s in this study, although recent clinical trials and reports have used ablation time of 8 to 12 s [5–7, 14]. This is because pigs are more prone to stenosis than humans after endoscopic resection or ablation [15, 16]. Moreover, previous reports of cryoablation in pigs and humans have shown that pigs have deeper and more severe injuries than do humans [17]. Based on these previous reports, the ablation time was restricted to 8 s.

There are four major ablation devices that can be used for esophageal neoplastic lesions in the clinical scenario: photodynamic therapy (PDT), argon plasma coagulation (APC), RFA, and cryoablation. PDT has shown favorable antitumor effects. However, it requires the intravenous administration of a photosensitizer drug and has additional side effects, such as phototoxicity, esophageal stricture, and severe pain [18]. APC is a noncontact electrocoagulation technique that uses argon gas for ablation, and it can only treat small residual or recurrent areas following EMR, ESD, or RFA [19]. Moreover, in spray cryoablation, cryogenic fluids are applied over the esophageal mucosa by using a spray catheter, it requires large equipment, and it is difficult to control to target specific areas in superficial lesions. Currently, RFA has the highest amount of supporting evidence for clinical application, and has been recommended as the standard ablation technique, especially for Barrett’s neoplasia, by several national and international associations for endoscopy [20, 21]. However, there are some obstacles must be overcome in RFA for ESCC with regard to the durability of the treatment effect [22]. CbFAS is a simple and effective procedure for superficial ESCC as well as Barret’s neoplasia, and it is associated with less post-procedural pain than is RFA [9]. Therefore, we believe this to be a key advantage of CbFAS and evaluated the feasibility of the procedure both before and after ER, which constitute the most important unmet clinical needs in the management for patients with ESCC. In particular, CbFAS for EMR scar is considered to be a treatment option for residual lesions after ER, and EMR + CbFAS is considered to be a treatment option when residual lesions are suspected at vertical or lateral margins during ER. According to the results of this study, we can carefully attempt to evaluate the safety and efficacy of this combination strategy for theselesions in a clinical trial.

This study has some limitations. First, endoscopic resection was performed using EMR. Therefore, treatment for large lesions was not investigated. In additon, we only used small numbers of animal model and could not evaluate the antitumor effect for neoplastic lesions. Further study is needed to increase number of cases.　While both tissue thickness and fragility differ between humans and animal models, we will evaluate the direct effects as well as safety of the combination of CbFAS with ER for neoplastic lesions in a carefully designed clinical trial.

Combination treatment with ER and CbFAS is technically feasible and did not differ from CbFAS alone with regard to tissue damage. Thus, this combination treatment strategy offers a therapeutic option for the management of metachronous and large SESCCs.

## Supplementary Information

**Additional file 1.** Simultaneous use of endoscopic mucosal resection (EMR) and Cryoballoon focal ablation system (CbFAS). CbFAS was performed immediately after EMR for a post-EMR mucosal defect. There was no obvious stenosis at day32 after treatment.

## Data Availability

All data generated or analyzed during this study are included in this published article.

## Abbreviations

APC: Argon plasma coagulation
CbFAS: Cryoballoon focal ablation system
EMR: Endoscopic mucosal resection
ER: Endoscopic Resection
ESD: Endoscopic submucosal dissection
RFA: Radiofrequency ablation
SESCC: Superficial esophageal squamous cell carcinoma
PDT: Photodynamic therapy
